# A mathematical model of the dynamics of prion aggregates with chaperone-mediated fragmentation

**DOI:** 10.1007/s00285-015-0921-0

**Published:** 2015-08-22

**Authors:** Jason K. Davis, Suzanne S. Sindi

**Affiliations:** University of California, 5200 N Lake Rd, Merced, 95343 USA

**Keywords:** Nucleated polymerization, Prions, Protein aggregation, Protein misfolding, Yeast, 92B05, 92C45

## Abstract

Prions are proteins most commonly associated with fatal neurodegenerative diseases in mammals but are also responsible for a number of harmless heritable phenotypes in yeast. These states arise when a misfolded form of a protein appears and, rather than be removed by cellular quality control mechanisms, persists. The misfolded prion protein forms aggregates and is capable of converting normally folded protein to the misfolded state through direct interaction between the two forms. The dominant mathematical model for prion aggregate dynamics has been the nucleated polymerization model (NPM) which considers the dynamics of only the normal protein and the aggregates. However, for yeast prions the molecular chaperone Hsp104 is essential for prion propagation. Further, although mammals do not express Hsp104, experimental assays have shown Hsp104 also interacts with mammalian prion aggregates. In this study, we generalize the NPM to account for molecular chaperones and develop what we call the enzyme-limited nucleated polymerization model (ELNPM). We discuss existence, uniqueness and stability of solutions to our model and demonstrate that the NPM represents a quasi-steady-state reduction of our model. We validate the ELNPM by demonstrating agreement with experimental results on the yeast prion $[$*PSI*${}^{+}]$ that could not be supported by the NPM. Finally, we demonstrate that, in contrast to the NPM, the ELNPM permits the coexistence of multiple prion strains.

**Electronic supplementary material:**

The online version of this article (doi:10.1007/s00285-015-0921-0) contains supplementary material, which is available to authorized users.

## Introduction

The central dogma of molecular biology stipulates that phenotypes, an organism’s expressed states, are determined by genotypes, the vertically transmitted DNA (Crick 1958). However, the link between genotype and phenotype is not always this direct. Today we understand that a number of phenotypes are determined epigenetically, without a change to the nucleotide sequence of DNA (Goldberg et al. 2007). In 1965, a number of yeast phenotypes were found to violate the laws of Mendelian inheritance and were thus inconsistent with DNA-based transmission (Cox et al. 1988). Further experimental studies demonstrated that the phenotypic states were not the function of the underlying DNA but were the function of a misfolded (prion) protein (Wickner 1994). As such, the phenotypes were transmitted by the proteins themselves. This phenomenon of “protein only inheritance,” also called the prion hypothesis, has over time gone from highly controversial to commonly accepted (Tuite and Serio 2010). Today, nearly a dozen proteins in yeast have been shown to be able to behave as prions (Liebman and Chernoff 2012). Of course, prions extend far beyond yeast. In mammals, prions are associated with a number of irreversible fatal neurological diseases such as Creutzfeldt–Jakob disease, fatal familial insomnia, chronic wasting disease and bovine spongiform encephalopathy. Mammalian prion diseases have varying modes of transmission and have been shown to be able to pass from one species to another. At present, all mammalian prion diseases are the result of a single protein, PrP (Aguzzi and Polymenidou 2004). In addition, prion diseases are closely related to other protein misfolding diseases such as Parkinson’s, Huntington’s, Alzheimer’s and diseases (Brundin et al. 2010; Knowles et al. 2014; Brettschneider et al. 2015).

Although humans are vastly different from yeast, the dynamics of prion proteins in both hosts is quite similar. Both mammals and yeast have cellular machinery dedicated to identifying and removing misfolded proteins (Nelson et al. 2008)—prion proteins are capable of evading such protective mechanisms and transmitting their misfolded (prion) state to other normally folded proteins. Prion proteins aggregate into complexes which act as templates for initiating further misfolding of normally folded protein. These aggregated complexes may also fragment into smaller units, each of which can template further misfolding (Sindi and Serio 2009; Tuite and Serio 2010). Finally, in order to spread the prion state to a colony or throughout a tissue, prion aggregates must be transmitted to other cells. In yeast colonies, prion aggregates are transmitted from mother to daughter cells during cell division (Tuite and Cox 2003; Derdowski et al. 2010). In mammalian prion diseases, PrP aggregates are thought to be transmitted extracellularly (Collinge 2001). For other mammalian neurodegenerative diseases, there is a growing body of evidence suggesting neuron to neuron propagation of the misfolded proteins (Brettschneider et al. 2015).

Many mathematical models have been developed to study the dynamics of prion aggregates primarily in the context of the mammalian host (Masel et al. 1999; Prüss et al. 2006; Greer et al. 2006; Calvez et al. 2010). Tanaka et al. (2006) applied these mathematical models to the $[$*PSI*${}^{+}]$ prion in yeast. However, experimental studies of $[$*PSI*${}^{+}]$ have shown that the molecular chaperone Hsp104 is essential for fragmentation, and recent studies have demonstrated that Hsp104 acts in a rate limiting fashion with respect to fragmentation (Satpute-Krishnan et al. 2007; Derdowski et al. 2010). We also note that fragmentation is important not only for efficient conversion of normal protein by providing more templates, but also to ensure there are sufficiently many templates to allow efficient transmission; thus an accurate model of fragmentation is essential to understanding the in vivo dynamics of prion aggregates. As such, to accurately model prion aggregates in yeast, the dynamics of Hsp104 and its interaction with prion aggregates must be considered.

Further, modeling Hsp104 will lend insight to more general prion and protein misfolding disorders. Although no chaperones are known to be involved in mammalian prion dynamics, prion amplification in mammals necessarily requires aggregate fragmentation (Masel et al. 1999). In addition, while mammals do not express Hsp104, recent in vitro work demonstrates that engineered mutants of Hsp104 suppress the toxicity of misfolded protein aggregates associated with mammalian neurodegenerative disorders (Jackrel and Shorter 2014). At present, no mathematical model exists which considers the dynamics of protein aggregates in the presence of a chaperone mediating fragmentation.

In this study we develop a mathematical model of prion dynamics where fragmentation requires the interaction of Hsp104 with aggregated proteins. In Sects. 2 and 3 we provide the mathematical background and analysis of our model, which we call the enzyme-limited nucleated polymerization model (ELNPM). In Sect. 4 we illustrate the necessity of including enzyme-limited fragmentation by demonstrating important experimental properties of $[$*PSI*${}^{+}]$ that are not described by previously published mathematical models. We also demonstrate that in contrast to models which consider only the prion aggregates, interactions with the enzyme Hsp104 permit stable co-existence of multiple prion strains. In Sect. 5 we provide a summary and concluding remarks.

## Mathematical models of prion aggregate fragmentation

We develop our model of enzyme-mediated fragmentation by considering the key biochemical processes involved in the dynamics of prions. We first discuss the dynamics included in previous mathematical formulations and then detail the additional features necessary to depict interactions between enzymes and aggregates. Finally, we demonstrate that through a series of assumptions, consistent with the yeast prion $[$*PSI*${}^{+}]$, our system of infinite ordinary differential equations can be analyzed with a 5-dimensional system of differential equations which approximates the full system dynamics.

### Prion aggregate dynamics

**Fig. 1:**
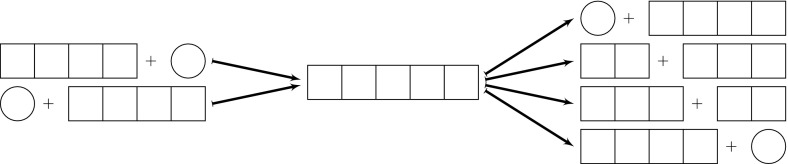
Nucleated polymerization model: conversion and fragmentation ($n_0=2$). Conversion of healthy protein (*circles*) lengthens the aggregate (*squares*), which may in turn fragment. If a daughter fragment is smaller than the stable nucleus size ($n_0$), it is immediately disassociated into healthy protein monomers

While the biochemical processes depicted differs between prion model formulations (Masel et al. 1999; Prüss et al. 2006; Greer et al. 2006; Calvez et al. 2010), in all cases aggregates change size through conversion and fragmentation. That is, a prion aggregate increases in length by actively converting and incorporating normal (healthy) protein monomers. Typically, aggregates are assumed to be linear fibrils and, as such, conversion of normal protein can only take place on one of the fibril ends. Aggregates may also fragment into two smaller aggregates, each of which now act as a template to convert additional protein. It is often assumed that any aggregates smaller than the minimum stable size, $n_0$, immediately disassociate into healthy prion monomers (see Fig. 1).

Such models are referred to as nucleated polymerization models (NPM); mathematical formulations of the NPM were first introduced and subsequently validated by Nowak et al. (1998) and Masel et al. (1999). This model is so-named due to the assumption that there is a minimum “stable” size of a prion aggregate (a nucleus). The spontaneous formation of such an initial nucleus (or seed, as it is also called) is the time-limiting step in prion disease initialization, but once seeded, the disease progresses primarily by the processes of conversion, fragmentation and transmission.

The NPM equations are derived from the Law of Mass Action applied to a minimal set of kinetic rate equations. Masel et al. (1999) give them as

1 $$\begin{aligned} s'&= \alpha _s - \mu _s s(t) - 2\beta s(t) \sum _{i=n_0}^\infty u_i(t) + \gamma (n_0-1)n_0 \sum _{i=n_0}^\infty u_i(t), \end{aligned}$$

2 $$\begin{aligned} u_m'&= -2\beta s(t)[u_m(t)-u_{m-1}(t)] - [\mu _0+\gamma (m-1)]u_m(t) + 2\gamma \sum _{i=m+1}^\infty u_i(t), \end{aligned}$$

where *s*(*t*) denotes the concentration of healthy protein and $u_m(t)$ the density of aggregates of size *m*. Many authors (Greer et al. 2006; Prüss et al. 2006; Engler et al. 2006) have studied the more analytically-tractable equations that come from a continuous relaxation of aggregate sizes:

3 $$\begin{aligned} s'&= \alpha _s - \mu _s s(t) - 2\beta s(t) \int _{x_0}^\infty u(t,x)dx + \gamma {x_0}^2 \int _{x_0}^\infty u(t,x)dx, \end{aligned}$$

4 $$\begin{aligned} \frac{\partial u}{\partial t}&= -2\beta s(t) \frac{\partial u}{\partial x} - [\mu _0+\gamma x]u(t,x) + 2\gamma \int _x^\infty u(t,y)\,dy. \end{aligned}$$

Though it is known that this latter system converges weakly to the former in the limit of large average aggregate size under very general assumptions (Doumic et al. 2009; Doumic and Gabriel 2010), we choose to generalize the discrete model for simplicity.

In adapting this prion model to yeast, we identify the kinetic parameters as representing the following physical quantities:

- $\alpha _s$ the basal rate of transcription of Sup35,
- $\mu _s$ the decay (or dilution) rate of Sup35,
- $n_0$ the minimum stable aggregate size (aggregates of size smaller than $n_0$ immediately disassociate into soluble Sup35),
- $\mu _0$ the decay (or dilution) rate of aggregated protein,
- $\beta$ the rate of conversion of healthy protein (from the end of a prion filament), and
- $\gamma (m-1)$ the fragmentation rate of a prion aggregate of size *m*.

### Enzyme-mediated fragmentation

We now draw attention to an underlying assumption in these equations that we will modify: for the mammalian prion PrP, it was assumed that the fragmentation rate is an intrinsic function of the aggregate size itself. However, with yeast prion systems, it has been demonstrated that fragmentation requires the additional presence of heat-shock protein 104 (Hsp104) (Satpute-Krishnan et al. 2007). Its under- or over-expression can eliminate prion aggregates entirely (Chernoff et al. 1995). Additionally, over-expressing Sup35 results in a translational shift in the aggregate-size density (Derdowski et al. 2010)—the model equations of Masel et al. (1999) do not admit such behavior (see Sect. 4.3), suggesting the possibility of a rate-limited fragmentation mechanism rooted in the Hsp104 interactions.

**Fig. 2:**
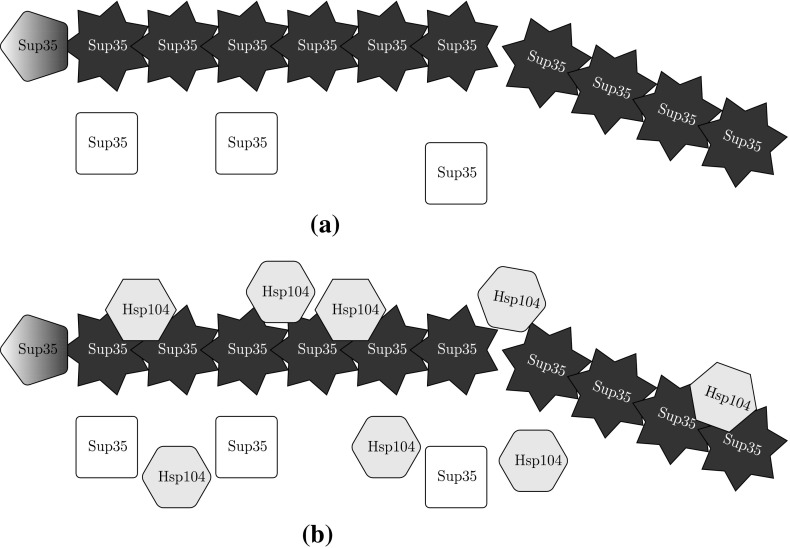
NPM is based on a random breaking of the prion aggregate (*stars*), while our model introduces the Hsp104 enzyme (*hexagons*) that mechanistically fragments the aggregate. Healthy Sup35 (*squares*) are converted by the ends of the prion aggregate in both models; proteins undergoing conversion are represented with *pentagons* to demonstrate the active change in conformation. **a** Nucleated polymerization model (stochastic fragmentation) **b** enzyme-limited polymerization model (mechanistic fragmentation via Hsp104 chaperone)

Though yeast prion systems have been studied with the NPM (Tanaka et al. 2006), we believe the impact of Hsp104 to be nonnegligible and explicitly consider the Hsp104 concentration and dynamics. We assume a prion aggregate of size *i* has $i-1$ sites to which a hexamer (the active unit) of Hsp104 can bind and subsequently fragment—we denote such an aggregate with *j* bound hexamers as $X_{i,j}$. We note that there are actually $$\left( {\begin{array}{c}i-1\\ j\end{array}}\right) $$ unique configurations of bound Hsp104 that $X_{i,j}$ could refer to, but the proposed kinetic equations will best be described by the amount of Hsp104 bound, not their configuration. We use standard terminology for the enzyme kinetics (parameters $k_{\text {on}}$ and $k_{\text {off}}$), and for simplicity, we do not model the formation of Hsp104 hexamers from monomers explicitly. We additionally define $\alpha _h$ and $\mu _h$ for Hsp104 similarly as $\alpha _s$ and $\mu _s$ for Sup35. Lastly, while we write the dilution rates $\mu _s$, $\mu _h$, and $\mu _0$ separately, we will take them as all equal to the rate of growth of the yeast cell in our numerical experimentation. We now propose our generalization in the form of the following kinetic relations (and illustrate in Fig. 2).

Translation and Dilution
Aggregation (Polymerization/Coagulation): $$\begin{aligned} \text {Sup35}+X_{i,j} \xrightarrow [2\beta ]{} X_{i+1,j},\qquad \end{aligned}$$
Enzyme Kinetics
Fragmentation (with unspecified probability $\displaystyle \kappa (m,n;i,j)$)
  $$\begin{aligned} X_{i,j} \xrightarrow [\gamma j]{} {\left\{ \begin{array}{ll} X_{m,n} + \text {Hsp104} + X_{i-m,j-n-1} &{}\quad m,i-m \ge n_0 \\ m\text {Sup35} + (n+1)\text {Hsp104} + X_{i-m,j-n-1} &{}\quad m < n_0, i-m \ge n_0 \\ X_{m,n} + (j-n)\text {Hsp104} + (i-m)\text {Sup35} &{}\quad m \ge n_0, i-m < n_0 \\ i\text {Sup35} + j\text {Hsp104} &{}\quad m,i-m < n_0. \end{array}\right. } \end{aligned}$$

The key unknown in our model is the density of configurations of bound Hsp104, which is incorporated into the fragmentation kernel $\kappa (m,n;i,j)$. Typically, all fragmentation sites in an aggregate are taken to be equally likely (Masel et al. 1999; Prüss et al. 2006; Greer et al. 2006):

5 $$\begin{aligned} \sum _{n=0}^{m-(i-j)} \kappa (m,n;i,j) = \frac{1}{i-1}. \end{aligned}$$

Furthermore, we require total Sup35 and Hsp104 to be conserved across fragmentation events, which corresponds to

6 $$\begin{aligned} \sum _{m=1}^{i-1} \sum _{n=0}^{m-1} m \kappa (m,n;i,j)=\frac{i}{2} \quad \text {and} \quad \sum _{m=1}^{i-1} \sum _{n=0}^{m-1} n \kappa (m,n;i,j)=(j-1)/2. \end{aligned}$$

We claim that

7 $$\begin{aligned} \kappa (m,n;i,j) = \frac{1}{i-1} \frac{\left( {\begin{array}{c}m-1\\ n\end{array}}\right) \left( {\begin{array}{c}i-m-1\\ j-n-1\end{array}}\right) }{\left( {\begin{array}{c}i-2\\ j-1\end{array}}\right) } = \frac{1}{j} \frac{\left( {\begin{array}{c}m-1\\ n\end{array}}\right) \left( {\begin{array}{c}i-m-1\\ j-n-1\end{array}}\right) }{\left( {\begin{array}{c}i-1\\ j\end{array}}\right) } \end{aligned}$$

is the natural choice, which follows from taking each $$\left( {\begin{array}{c}i-1\\ j\end{array}}\right) $$ configuration of $X_{ij}$ to be equally likely, which in turn corresponds to enzyme binding acting on a faster time-scale than conversion and fragmentation (refer to the Supplemental Materials for the argument).

### Enzyme-limited nucleated polymerization model

With the biochemical processes defined, we are now able to formally derive our ELNPM. We define *s*(*t*) as the concentration of soluble Sup35, $\eta (t)$ as the concentration of aggregates, *z*(*t*) as the concentration of bound Sup35 ($z(t)\ge n_0\eta (t)$), *h*(*t*) as the concentration of unbound Hsp104, and $z_b(t)$ as the concentration of bound Hsp104. Using [*S*] to denote the concentration of chemical species *S*, these definitions correspond to $s(t)=[\text {Sup35}](t)$, $h(t)=[\text {Hsp104}](t)$, and

8 $$\begin{aligned} \eta (t)=\sum _{i=n_0}^\infty \sum _{j=0}^{i-1} u_{ij}(t), \quad z(t)=\sum _{i=n_0}^\infty \sum _{j=0}^{i-1} iu_{ij}(t), \quad z_b(t)=\sum _{i=n_0}^\infty \sum _{j=0}^{i-1}ju_{ij}(t), \end{aligned}$$

where we have let $u_{ij}(t)=[X_{ij}](t)$. Let us define a new quantity *p*(*t*) by the relation $z_b(t)=p(t)[z(t)-\eta (t)]$ and apply the Law of Mass Action to our proposed kinetic equations. We obtain

9a $$\begin{aligned} s'(t)&= \alpha _s - \mu _s s(t) - 2\beta s(t) \eta (t) + \gamma (n_0-1)n_0 \eta (t) \sum _{i=n_0}^\infty \sum _{j=0}^{i-1} \frac{j}{i-1} \frac{u_{ij}(t)}{\eta (t)} \end{aligned}$$

9b $$\begin{aligned} h'(t)&= \alpha _h - \mu _h h(t) + [(k_{\text {off}}+\gamma )p(t) - k_{\text {on}}h(t)(1-p(t))] (z(t)-\eta (t)) \nonumber \\&\quad + \gamma (n_0-1)(n_0-2)\eta (t) \sum _{i=n_0}^\infty \sum _{j=0}^{i-1} \frac{j(j-1)}{(i-1)(i-2)} \frac{u_{ij}(t)}{\eta (t)} \end{aligned}$$

9c $$\begin{aligned} \eta '(t)&= -(\mu _0+\gamma p(t))\eta (t) + \gamma p(t) z(t) - 2\gamma (n_0-1) \eta (t) \sum _{i=n_0}^\infty \sum _{j=0}^{i-1} \frac{j}{i-1} \frac{u_{ij}(t)}{\eta (t)} \end{aligned}$$

9d $$\begin{aligned} z'(t)&= 2\beta s(t) \eta (t) - \mu _0 z(t) - \gamma n_0 (n_0-1) \eta (t) \sum _{i=n_0}^\infty \sum _{j=0}^{i-1} \frac{j}{i-1} \frac{u_{ij}(t)}{\eta (t)} \end{aligned}$$

9e $$\begin{aligned} p'(t)&= k_{\text {on}}h(t)(1-p(t)) - (k_{\text {off}}+\gamma )p(t) + p(t)^2 - \frac{2\beta s(t) \eta (t) p(t)}{z(t)-\eta (t)} \nonumber \\&\quad + \frac{\gamma (n_0-1)(n_0-2)\eta (t)}{z(t)-\eta (t)}\left( \sum _{i=n_0}^\infty \sum _{j=0}^{i-1}\frac{j}{i-1} \frac{u_{ij}(t)}{\eta (t)} \right. \nonumber \\&\quad \left. - \frac{1}{p(t)}\sum _{i=n_0}^\infty \sum _{j=0}^{i-1} \frac{j(j-1)}{(i-1)(i-2)}\frac{u_{ij}(t)}{\eta (t)}\right) . \end{aligned}$$

We note that $u_{ij}/\eta$ defines a probability mass function for the joint random variable (*I*, *J*) over $\{(i,j): 0 \le j < i, i \ge n_0\}$, thus the sums may be interpreted as an expected value. In the interest of obtaining a simple set of equations, we approximate

10 $$\begin{aligned} {\mathbb {E}}\left[ \frac{J}{I-1}\right] \approx {\mathbb {E}}[J] / {\mathbb {E}}[I-1] = z_b/(z-\eta ) = p \end{aligned}$$

and

11 $$\begin{aligned} {\mathbb {E}}\left[ \frac{J(J-1)}{(I-1)(I-2)}\right] \approx {\mathbb {E}}[J]^2 / {\mathbb {E}}[I-1]^2 = p^2. \end{aligned}$$

The error of this approximation is on the order of the inverse square of the average aggregate size, a size which is typically large by assumption (Prüss et al. 2006; Greer et al. 2006; Doumic et al. 2009). (This analysis is provided in the Supplemental Materials.)

**Fig. 3:**
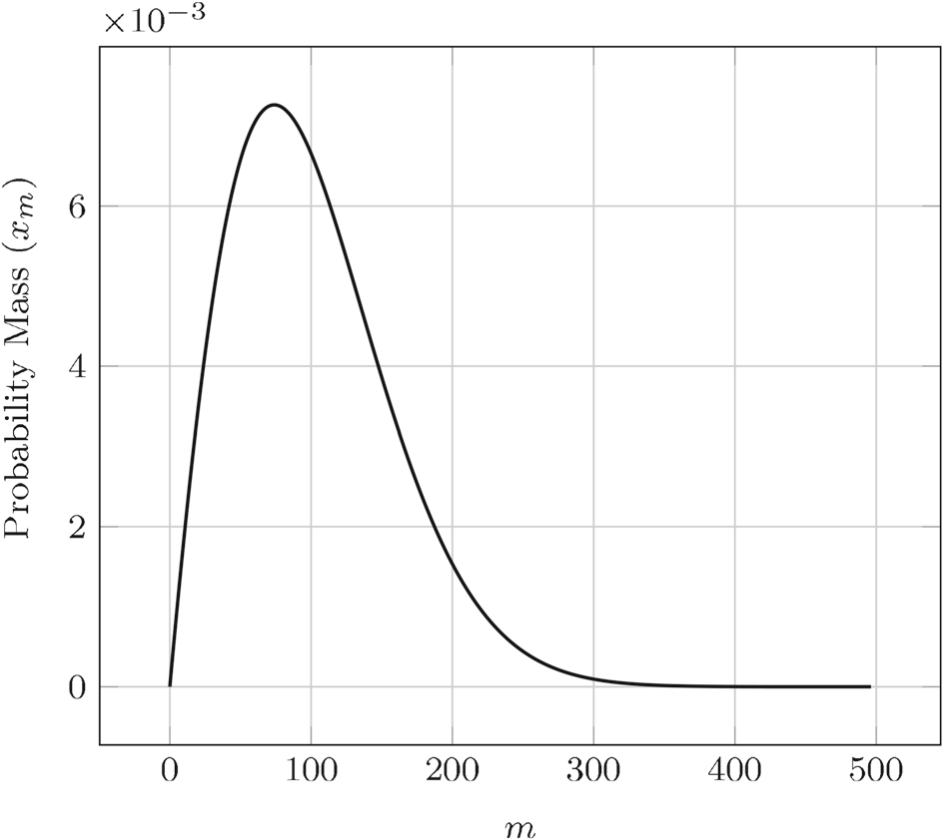
Plot of steady-state $x_m=u_{m+n_0-1}/\eta$ with parameter values chosen from Tanaka et al. (2006), appropriately modified to match the steady-state, effective fragmentation rate with the paper’s constant rate

Making these substitutions, we obtain the enzyme-limited, nucleated polymerization model (ELNPM):

12a $$\begin{aligned} s'(t)&= \alpha _s - \mu _s s(t) - 2\beta s(t) \eta (t) + \gamma p(t) (n_0-1)n_0 \eta (t) \end{aligned}$$

12b $$\begin{aligned} h'(t)&= \alpha _h - \mu _h h(t) + [(k_{\text {off}}+\gamma )p(t) - k_{\text {on}}h(t)(1-p(t))] (z(t)-\eta (t)) \end{aligned}$$

12c $$\begin{aligned} \eta '(t)&= -[\mu _0+\gamma p(t)(2n_0-1)]\eta (t) + \gamma p(t) z(t) \end{aligned}$$

12d $$\begin{aligned} z'(t)&= 2\beta s(t) \eta (t) - \mu _0 z(t) - \gamma p(t) n_0 (n_0-1) \eta (t) \end{aligned}$$

12e $$\begin{aligned} p'(t)&= k_{\text {on}}h(t)(1-p(t)) - (k_{\text {off}}+\gamma )p(t) - p(t)\left( \frac{2\beta s(t) \eta (t)}{z(t)-\eta (t)} - \gamma p(t)\right) , \end{aligned}$$

and marginal density equations

13 $$\begin{aligned} \frac{du_m}{dt} = -2\beta s(t)[u_m(t)-u_{m-1}(t)] - [\mu +\gamma p(t)(m-1)]u_m + 2\gamma p(t) \sum _{i=m+1}^\infty u_i(t). \end{aligned}$$

We note that these equations may alternatively be derived by prescribing the binomial form $$u_{ij}(t)=\left( {\begin{array}{c}i-1\\ j\end{array}}\right) p(t)^j[1-p(t)]^{i-j-1}u_i(t)$$, then finding the unique *p*(*t*) that preserves Hsp104 conservation across fragmentation events.

We further observe that the systems $(s,\{u_m\})$ and $(s,\eta ,z)$ are identical to that of the original NPM (Masel et al. 1999), except the constant fragmentation rate $\gamma$ has been replaced by the time-varying quantity $\gamma p(t)$. This provides the interpretation of *p*(*t*) as the measure of how effectively the system is fragmenting at a given time. Furthermore, this formulation suggests a quasi-steady-state interpretation of our approximation, since now every aggregate is always bound with Hsp104 proportionally as *p*(*t*); that is, the enzyme binding reaches equilibrium before any conversion or fragmentation events occur. We finally note that the first 2 terms in Eq. (12e) reflect a Michaelis–Menten simplification of the enzyme kinetics (Segel and Slemrod 1989); however, since Hsp104 off-binding results in a change in the amount of binding substrate with probability $\gamma /(\gamma +k_{\text {off}})$, we may view the last term as the correction to preserve Hsp104 conservation.

Before detailed analysis of the ELNPM, we briefly examine the qualitative form of the aggregate size distribution. In Fig. 3 we plot a typical equilibrium solution to Eq. (13), where we have defined $x_m=u_{n_0-1+m}/\eta$ to be the corresponding probability mass function over the natural numbers. As expected, given the asymptotic similarity of our system to (Masel et al. 1999) (in that $\gamma p(t)$ presumably converges to a fixed $\tilde{\gamma }$), the equilibrium size density is of the same distribution.

## Analysis of the ELNPM

We first prove a few results on existence and uniqueness for this system and then provide a non-dimensionalized, transformed system we will use to study stability. We analytically demonstrate the stability of the disease-free state and derive conditions which will ensure aggregate persistence.

### Existence and uniqueness of solutions

#### **Theorem 1**

Trajectories of Eq. () remain invariant under a bounded, “feasible” subset of the non-negative cone ${\mathbb {R}}^5_+$.

#### *Proof*

Let us define the feasible subset to be the set of all $(s,h,\eta ,z,p)$ where $s,h,\eta ,z \ge 0$ and $0 \le p \le 1$, with the further restriction that $z \ge n_0\eta$, $0 \le s+z \le \alpha _s/\mu _0$, and $0 \le h+p(z-\eta ) \le \alpha _h/\mu _0$. For analytical convenience, and in line with typical parameter regimes, we assume $\mu _0 \le \mu _s, \mu _h$ and $\frac{\gamma }{2\beta } < \frac{\alpha _s}{\mu _s}$.

Now consider the violation of any single constraint. It is straight-forward to show $s'\big |_{s<0}, h'\big |_{h<0}, \eta '\big |_{\eta <0},p'\big |_{p<0} \ge 0$. Similarly, $p'\big |_{p>1} \le 0$. Next, $(z-n_0\eta )'\big |_{z<n_0\eta }= 2\beta s \eta - \mu _0 (z-n_0\eta ) - n_0 \gamma p (z-n_0\eta ) \ge 0$, which also demonstrates the non-negativity of *z* since $z \ge n_0\eta \ge 0$. Writing $\mu _s/\mu _0=1+\epsilon /\mu _0$, we have $(s+z)'\big |_{s+z>\alpha _s/\mu _0}=\alpha _s-\mu _0(s+z)-\epsilon s \le 0$. With a similar approach, we also find $(h+p(z-\eta ))'\big |_{h+p(z-\eta )>\alpha _h/\mu _0} \le 0$. $\square$

#### **Theorem 2**

Solutions satisfy the existence and uniqueness criteria within the invariant, feasible region.

#### *Proof*

With the exception of the $\frac{\eta }{z-\eta }$ term in $p'$, the derivatives are polynomial in the dependent variables, which yields continuous partial derivatives. Considering now this last term, $z \ge n_0\eta$ in our feasible region so we have $0 \le \frac{\eta }{z-\eta } \le \frac{1}{n_0-1} \le 1$. This term’s partial derivatives are also continuous in this region; let $q(\eta ,z)=\eta /(z-\eta )$. Then, $z/\eta =1+1/q$ and

14 $$\begin{aligned} \begin{aligned} q_\eta&= \frac{\eta '}{z-\eta } + \frac{\eta (z-\eta )'}{(z-\eta )^2} = \frac{\eta '}{\eta } q + \frac{z'-\eta '}{\eta } q^2 \\&= q(q-1)\left( \mu _0+\gamma p (2n_0-1)-\gamma p (1/q+1)\right) \\&\quad + q^2\left( 2\beta s - \mu _0(1+1/q)-\gamma p n_0(n_0-1)\right) \\&= (q-1)\left( q\mu _0+q\gamma p(2n_0-1)-\gamma p (q+1)\right) \\&\quad + q\left( 2q\beta s - \mu _0(1+q)-q\gamma p n_0(n_0-1)\right) , \end{aligned} \end{aligned}$$

and

15 $$\begin{aligned} \begin{aligned} q_z&= -\frac{\eta }{(z-\eta )^2} z' = -q^2 \frac{z'}{\eta } = -q^2 \left( 2\beta s - \mu _0(1+1/q)-\gamma p n_0(n_0-1)\right) \\&= -q \left( 2q\beta s - \mu _0(1+q)-q\gamma p n_0(n_0-1)\right) . \end{aligned} \end{aligned}$$

Thus, $q, q_\eta ,q_z$ are continuous in our region, even as $\eta \rightarrow 0^+$. This establishes existence and uniqueness. $\square$

### Non-dimensionalized equations

We now reduce the ELNPM equations to non-dimensional form,

16a $$\begin{aligned} s'&= A_s(1-s) - Bs\eta + (n_0-1)n_0 p\eta \end{aligned}$$

16b $$\begin{aligned} h'&= A_h(1-h) + r[(\omega +n_0-1)\left( k_{-1}p- k_{1} h[1-p]\right) + (n_0-1)(n_0-2) p^2]\eta \end{aligned}$$

16c $$\begin{aligned} \eta '&= \left( \omega - A_0/p - n_0 + 1\right) p\eta \end{aligned}$$

16d $$\begin{aligned} \omega '&= B s - p(\omega +1)\omega \end{aligned}$$

16e $$\begin{aligned} p'&= k_{1}h(1-p) - k_{-1}p - p\left( \frac{Bs}{\omega +n_0-1}-p\right) \end{aligned}$$

where we have replaced *z*(*t*) by the displacement of the average aggregate size from the minimum size $\omega (t)=z(t)/\eta (t)-n_0 \ge 0$. We have scaled time by $\gamma$, *s*(*t*) and $\eta (t)$ by $\alpha _s/\mu _s$ and *h*(*t*) by $\alpha _h/\mu _h$, and used the following non-dimensional constants

17 $$\begin{aligned} A_s= & {} \mu _s/\gamma , \quad A_h = \mu _h/\gamma , \quad A_0 = \mu _0/\gamma , \quad B = \frac{2\alpha _s \beta }{\gamma \mu _s}, \nonumber \\ k_{-1}= & {} (k_{\text {off}}+\gamma ) / \gamma , \quad k_{1} = \frac{k_{\text {on}} \alpha _h}{\gamma \mu _h}, \quad r = \frac{\alpha _s / \mu _s}{\alpha _h / \mu _h}. \end{aligned}$$

All subsequent analysis will done with respect to these non-dimensional equations. We note that by construction $k_{-1}>1$ and by assumption, $B>1$.

### Asymptotic behavior of ELNPM

With the nondimensional equations established, we next consider the asymptotic behavior of the ELNPM. We call any trajectory satisfying $\lim _{t\rightarrow \infty }\eta (t)=0$*disease-free*; otherwise, we call the prion state *persistent*. Prüss et al. (2006) observed that an appropriate transformation could reduce the NPM equations to the standard SEIS model of mathematical epidemiology—a model which is governed entirely by a single parameter $R_0$ (the basic reproductive number). If $R_0<1$, the only equilibrium is disease-free and is globally stable. If $R_0>1$, a unique endemic equilibrium appears and exchanges stability with the disease-free state; that is, the endemic equilibrium is asymptotically globally stable (Li et al. 1999). Applying the transformation from Prüss et al. (2006), our $R_0$ will vary in time through its dependence on *p*(*t*):

18 $$\begin{aligned} R_0(p) = \frac{B/p}{(A_0/p+n_0-1)(A_0/p+n_0)}. \end{aligned}$$

It is convenient to think of the quantity $R_0(p(t))=R_t$ as the *effective reproductive number* of the disease system, where typically $R_t < R_0$. Though *p*(*t*) appears to always converge to a fixed steady-state value, it does so in a non-trivial way making it difficult to provide a Lyapunov analysis. Instead, we provide a local analysis of the disease-free state and offer numerical evidence in support of $R_0(p_{\text {disease-free}})$ determining global stability.

#### Disease-free steady state

At a disease-free equilibrium, we have $\eta =0$ and subsequently, $s=h=1$. Thus, we need only study solutions to

19 $$\begin{aligned} \begin{aligned} 0&= B-p\omega (\omega +1) \\ 0&= k_1(1-p)-k_{-1}p+p^2 - \frac{Bp}{\omega +n_0-1}. \end{aligned} \end{aligned}$$

This system has five solutions in general, though we shall show there is only a single solution inside our feasible region.

##### **Theorem 3**

There is a unique solution to Eq. (19) in our feasible region of trajectories.

##### *Proof*

We may write $p=p(\omega )=B/[\omega (\omega +1)]$; then $\omega$ satisfies

20 $$\begin{aligned} \begin{aligned} 0 = f(\omega )&= B^2(n_0-1) - B(n_0-1)(k_1+k_{-1})\omega \\&\quad - [B^2+Bk_{-1}n_0+\{1+(B-1)n_0\}k_1]\omega ^2 \\&\quad [-B(k_1+k_{-1})+k_1(2n_0-1)]\omega ^3 + k_1(n_0+1)\omega ^4 + k_1\omega ^5. \end{aligned} \end{aligned}$$

Since $\frac{k_1}{k_1+k_{-1}}<1<B$, we observe 2 sign changes in the coefficients of $f(\omega )$, implying 0 or 2 real roots by Budan’s theorem (Akritas 1982). We may also write $\omega =\omega (p)=-1/2+\sqrt{1/4+B/p}$. Since $p<1$, we have $\omega >\omega _{\text {min}}=-1/2+\sqrt{1/4+B}$. However, $f(0)=B^2(n_0-1)>0$, $f(\omega _{\text {min}})=-\frac{B^2}{2}(2B+(k_{-1}-1)(2n_0-3+ \sqrt{1+4B}))<0$, and $\lim _{\omega \rightarrow \infty }f(\omega )>0$. Thus, by sign analysis we have an infeasible root $\omega \in (0,\omega _{\text {min}})$ and a feasible root $\omega > \omega _{\text {min}}$. $\square$

We now establish local stability criteria of this root; let $p_0$ and $\omega _0$ be the unique solution to Eq. (19).

##### **Theorem 4**

The unique disease-free equilibrium is locally stable when $R_0(p_0)<1$ and unstable when $R_0(p_0)>1$.

##### *Proof*

The eigenvalues of the localized Jacobian will satisfy

21 $$\begin{aligned} 0= & {} (A_s+\lambda )(A_h+\lambda )(p_0(\omega _0+n_0-1)-A_0-\lambda ) \nonumber \\&\times \left[ \begin{array}{ll} \lambda ^2 &{}+ \left( 2\omega _0 p_0 + k_1 + k_{-1}-p_0 + \frac{B}{\omega _0+n_0-1}\right) \lambda \\ &{}\quad + p_0(1+2\omega _0)\left( k_{-1}+k_1-2p_0+\frac{B}{\omega _0+n_0-1}\right) + \frac{Bp_0\omega _0(\omega _0+1)}{(\omega _0+n_0-1)^2}. \end{array}\right] \nonumber \\ \end{aligned}$$

Of the five roots, the first 2 are clearly negative. The quadratic factor will also admit 2 stable roots: we see that its quadratic and linear coefficients are strictly positive and now show that the constant term is as well. If $k_1>0$, then $k_1+k_{-1}-2p_0>2(1-p_0)>0$. If not, then consider $p'$ at $p=k_1$:

22 $$\begin{aligned} p' < k_1(1-k_1)-k_{-1}k_1 + k_1^2 = k_1(1-k_{-1}) < 0. \end{aligned}$$

So, $p(t)<k_1$ for all time, implying $p_0<k_1$ and $k_1+k_{-1}-2p_0>k_{-1}-p_0>1-p_0>0$.

Finally, the remaining root will be negative when $p_0(\omega _0-n_0+1)-A < 0$. Substituting $\omega _0=-1/2+\sqrt{1/4+B/p_0}$ and simplifying, our expression reduces to

23 $$\begin{aligned} R_0(p_0) = \frac{B/p_0}{(A_0/p_0+n_0)(A_0/p_0+n_0-1)} < 1. \end{aligned}$$

$$\square $$

**Fig. 4:**
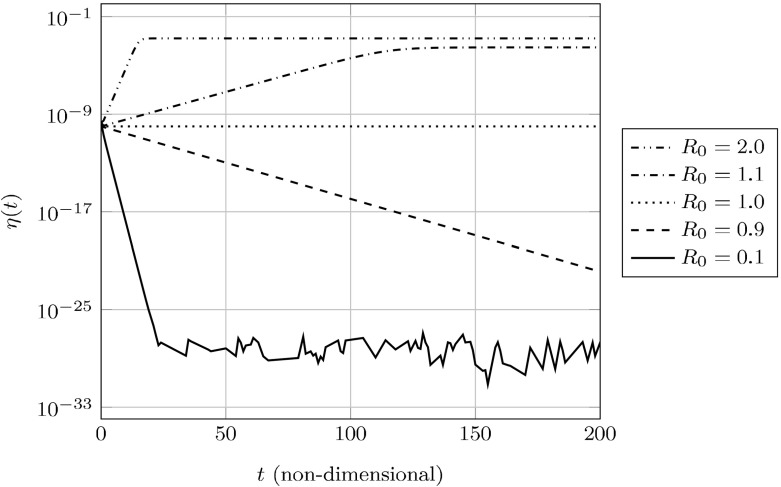
Non-dimensionalized plots of aggregate density over time with varying $R_0=R_0(p_0)$. The system is initialized with a $10^{-9}$ perturbation of aggregated protein from an otherwise healthy initial state

We support this claim numerically in Fig. 4, where we vary $R_0(p_0)$ across a range of parameters (described in Table 1). We observe that when $R_0(p_0) > 1$, there appears to be an attractive *endemic* equilibrium.

**Table 1:** A table of values used for the plots

Figures	$\alpha _h\,(\upmu {\mathrm {M}}\,{\mathrm {min}}^{-1})$	$\beta \,(\upmu {\mathrm {M}}^{-1}{\mathrm {min}}^{-1})$	$\gamma \,({\mathrm {min}}^{-1})$	$k_{\text {on}}\,(\upmu {\mathrm {M}}^{-1}{\mathrm {min}}^{-1})$	$k_{\text {off}}\,({\mathrm {min}}^{-1})$
3, 6 and 8	0.002567	6.0	0.00294	0.20000	2.00000
4 and 5	0.00142	–	0.00294	0.15964	2.93706
7	$4.16503\times 10^{-6}$	0.216456	0.0421667	81.8524	4.38533
8	–	0.216456	0.0421667	81.8524	4.38533
9a	0.002567	(6.0, 6.0)	(0.00294, 0.00294)	(0.2, –)	(2.0, –)
9b	0.002567	(6.0, 8.0)	(0.00294, 0.002)	(0.2, –)	(2.0, –)
10	0.002567	(6.0, 8.0)	(0.00294, 0.002)	(0.2, 0.21567)	(2.0, 2.0)

In all cases, $\alpha _s=0.0154\,\upmu {\mathrm {M}}\,{\mathrm {min}}^{-1}$, $n_0=n_1=n_2=\cdots =5$, and $\mu _s=\mu _h=\mu _0=\mu _1=\mu _2=\cdots =0.0077\,{\mathrm {min}}^{-1}$

#### Endemic steady state

The local instability of the disease-free equilibrium yields a persistent disease state; however, the numerical experimentation in Fig. 4 suggests further that there is an attractive, endemic equilibrium. Generally speaking, steady-state solutions to our system will be solutions of a quintic polynomial in five variables, thus preventing closed-form descriptions of such states. However, one can parameterize these values in terms of a fixed (but unknown) value $\tilde{p}$ corresponding to $p=\tilde{p}$:

24a $$\begin{aligned} \tilde{s}&= \frac{(A_0/\tilde{p}+n_0-1)(A_0/\tilde{p}+n_0)}{B/\tilde{p}} \end{aligned}$$

24b $$\begin{aligned} \tilde{h}&= 1- r(A_s/A_h)(1-\tilde{s}) \tilde{p}\left( 1-\frac{1}{A_0/\tilde{p}+2n_0-1}\right) \end{aligned}$$

24c $$\begin{aligned} \tilde{\eta }&= (A_s/A_0) \frac{1-\tilde{s}}{A_0/\tilde{p}+2n_0-1} \end{aligned}$$

24d $$\begin{aligned} \tilde{\omega }&= A_0/\tilde{p}+n_0-1, \end{aligned}$$

where $$\tilde{p}$$ satisfies

25 $$\begin{aligned} 0 = k_1 \tilde{h}(1-\tilde{p}) - k_{-1}\tilde{p} + \tilde{p}^2 - \frac{B\tilde{s}\tilde{p}}{\tilde{\omega }+n_0-1}. \end{aligned}$$

We note that this quadratic is uniquely invertible within our feasible region, which yields the recursive relation

26 $$\begin{aligned} \begin{aligned} 2\tilde{p}&= \left( k_{-1}+k_1\tilde{h}+\frac{B\tilde{s}}{\tilde{\omega }+n_0-1}\right) \\&\quad - \sqrt{\left( k_{-1}+k_1\tilde{h}+\frac{B\tilde{s}}{\tilde{\omega }+n_0-1}\right) ^2-4k_1\tilde{h}}. \end{aligned} \end{aligned}$$

Incidentally, if we suppose $k_1h+k_{-1} \gg \frac{B\tilde{s}}{\tilde{\omega }+n_0-1}, k_1h$, then we obtain

27 $$\begin{aligned} \tilde{p} = \frac{k_1h}{k_1h + k_{-1}} + O\left( \frac{1}{(k_1h+k_{-1})^2}\right) . \end{aligned}$$

We draw attention to the similarity between Eq. (27) and what would be the Michaelis–Menten value for $\tilde{p}$.

Our feasible region requires $s,h,\eta ,\omega >0$; $\tilde{s}$ and $\tilde{\omega }$ are always positive, while $\tilde{\eta }>0 \implies \tilde{s} < 1 \implies R_0(\tilde{p})>1$. This also gives us $\tilde{h}>0$, since we typically assume $\alpha _h \le \alpha _s \implies r(A_s/A_h) \le 1$, which implies

28 $$\begin{aligned} \tilde{h} >1-(1-\tilde{s})\tilde{p}\left( 1-\frac{1}{A_0/ \tilde{p}+2n_0-1}\right) > 0. \end{aligned}$$

Thus, we will have endemic equilibria when Eq. (26) has solutions satisfying $R_0(\tilde{p})>1$. Based on considerable numerical studies, we conjecture that this solution uniquely exists and is globally asymptotically stable when $R_0(p_0)>1$.

## Discussion

Formulating the ELNPM allows us to consider aspects of prion aggregate dynamics that cannot be explained by prior mathematical approaches that neglected the role of the Hsp104 chaperone in fragmentation. We first demonstrate that the NPM is a limiting case of the ELNPM and comment on implications of the ELNPM to the larger question of the appearance of prion strains in a population. We then demonstrate that the ELNPM is the first model capable of supporting two experimentally observed phenomena. First, the ELNPM is the first model capable of reproducing shifts in the aggregate densities associated with increases in synthesis of Sup35. Finally, we demonstrate that the binding kinetics of Hsp104 in the ELNPM allows the possibility of multiple co-existing prion strains. Intriguingly, the co-existence of multiple strains is thought to be crucial towards understanding the transmission of prion diseases between species (Chien et al. 2004).

### The NPM is a limiting case of ELNPM

Our moment-closed ELNPM model, Eq. (), is nearly identical to the original NPM model but with a time-varying, effective fragmentation rate $\gamma p(t)$ instead of the constant $\gamma$. When $p(t)\rightarrow \tilde{p}$, the dynamics of ELNPM will mirror that of the NPM with $\gamma$ replaced by $\gamma \tilde{p}$. As such, it is convenient to think of the NPM as a quasi-steady-state approximation of the full enzyme kinetics we have considered in our model. We informally used this observation in Sect. 3.3 to motivate (but not prove) global stability based on known results of the NPM system.

**Fig. 5:**
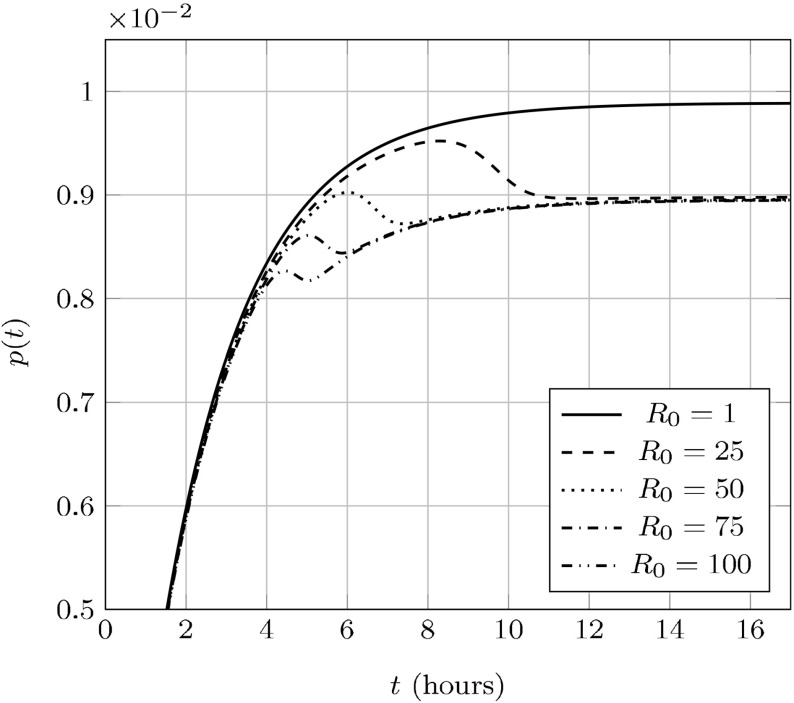
*p*(*t*) over time with varying $R_0=R_0(p_0)$; parameters are the same as in Fig. 4. The transient fragmentation efficiency may be higher (for small $R_0$) or lower (for large $R_0$) than the asymptotic efficiency

We plot in Fig. 5*p*(*t*) over the same parameters in Fig. 4 and note that—since yeast has a doubling time of roughly 90 min (Hartwell and Unger 1977) – *p*(*t*) will not reach its asymptotic value for a few cell-divisions. As such, even though the NPM represents a quasi-steady-state approximation it may not represent the aggregate dynamics during the early cell divisions following the introduction of prion aggregates.

### Transient fragmentation efficiency may impact prion stability

Although the NPM may be viewed as an asymptotic simplification of the ELNPM, we remark that the differences in transient behavior may provide insight into the underlying stochastic dynamics that arise when a single prion seed is introduced into a healthy yeast colony. The ELNPM predicts an initial fragmentation rate that can be larger or smaller than the asymptotic rate—this is because the availability of enzyme (Hsp104) is much larger than the availability of substrate (binding sites) in this initial configuration (see Fig. 5).

Since aggregate amplification is essential to spreading a prion disease these transient fragmentation rates may impact prion stability. For example, a higher transient fragmentation rate for a prion strain with low $R_0$ would represent a barrier to successful “seeding” of the prion state than would otherwise be predicted by a constant fragmentation rate. This provides a plausible mechanism for the removal of an initial prion aggregate appearing in a population and therefore the low frequency of spontaneous appearance of the prion state.

### Hsp104 acts as a rate limiter for fragmentation

We noted in Sect. 2 that the NPM does not admit translational shifts in the aggregate density as a function of increasing synthesis of the prion protein ($\alpha _s$), we now formally demonstrate this is the case. Let us revisit the quantity $u_m(t)$, the density of aggregates of size *m*. We write $x_m=u_{m+n_0-1}/\eta$—this quantity defines a probability mass function that is independent of the amount of aggregated protein. In our rescaled variables, we have

29 $$\begin{aligned} x_m' = -Bs(x_m-x_{m-1}) - p(m+\omega +1) x_m + 2p - 2p\sum _{i=0}^{m-1} x_i. \end{aligned}$$

Davis and Sindi (2015) gave a closed-form for $x_m$ at steady-state:

30 $$\begin{aligned} x_m = m(2\zeta +m-1) \frac{\varGamma (\zeta ^2)}{\varGamma (\zeta ^2+m+1)}\zeta ^m(\zeta -1)^{m-1}, \end{aligned}$$

where $$\zeta =\frac{\mu }{\gamma \tilde{p}}+n_0$$.

**Fig. 6:**
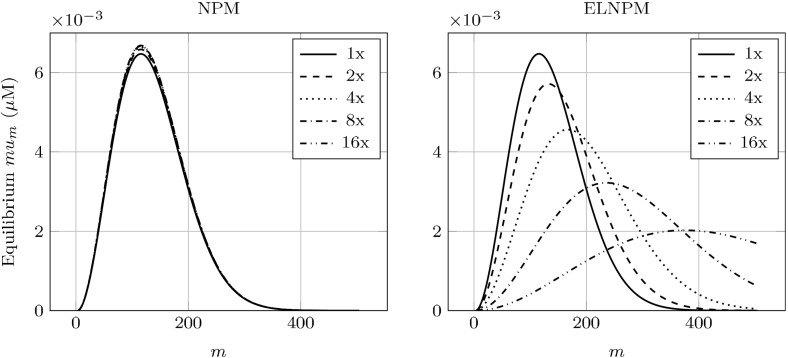
Theoretical shifts in the steady-state concentration of aggregate size distributions $\{mu_m\}$ by increasing the synthesis of normal protein ($\alpha _s$). *Left* $mu_m=\eta mx_{m-n_0+1}$ from the NPM. Though $x_m$ is invariant to $\alpha _s$, $\eta$ does have a dependency, resulting in the small changes in scaling. *Right* $mu_m$ from our ELNPM. Both the scaling and translation are affected by $\alpha _s$. Initial kinetic parameters are as in Fig. 3

The size distribution’s dependence on $\alpha _s$ can only occur through its relationship with the steady-state fragmentation efficiency $\tilde{p}$. This is fixed in the NPM, thus the size distribution will not change in response to changes in $\alpha _s$. This is in contradiction to the experimental results described by Derdowski et al. (2010). Since our model does allow $\tilde{p}$ to vary as a function of the kinetic parameters, we are able to numerically investigate qualitative shifts in the distribution. We demonstrate these shifts in Fig. 6, which are in qualitative agreement with the experiments of Derdowski et al. (2010). As such, experimental evidence supports that fragmentation can not be purely a function of the number of available fragmentation sites and must depend on the amount of Hsp104 in the system.

### Prion extinction and Hsp104 expression levels

Beyond translational shifts in aggregate size distribution, the $[$*PSI*${}^{+}]$ prion phenotype in yeast has been shown to be very sensitive to the amount of Hsp104 in the system (Higurashi et al. 2008; Doyle and Wickner 2009; Shorter and Lindquist 2008). Our mathematical formulation correctly captures the dependency of all prions to the under-expression of Hsp104. In contrast, and in agreement with recent experimental studies (Klaips et al. 2014), over-expression of Hsp104 does not necessarily drive prions to extinction.

First, sufficiently high concentrations of guanidine hydrochloride GdnHCl have been shown to severely disrupt the fragmentation process by inactivating Hsp104 (Ferreira et al. 2001; Byrne et al. 2007). Since fragmentation is halted the total number of aggregates will not change and aggregates will eventually be lost through dilution in the population due to cell division. Over time the population will be cured of the prion disease as the fraction of cells with aggregates approaches zero.

**Fig. 7:**
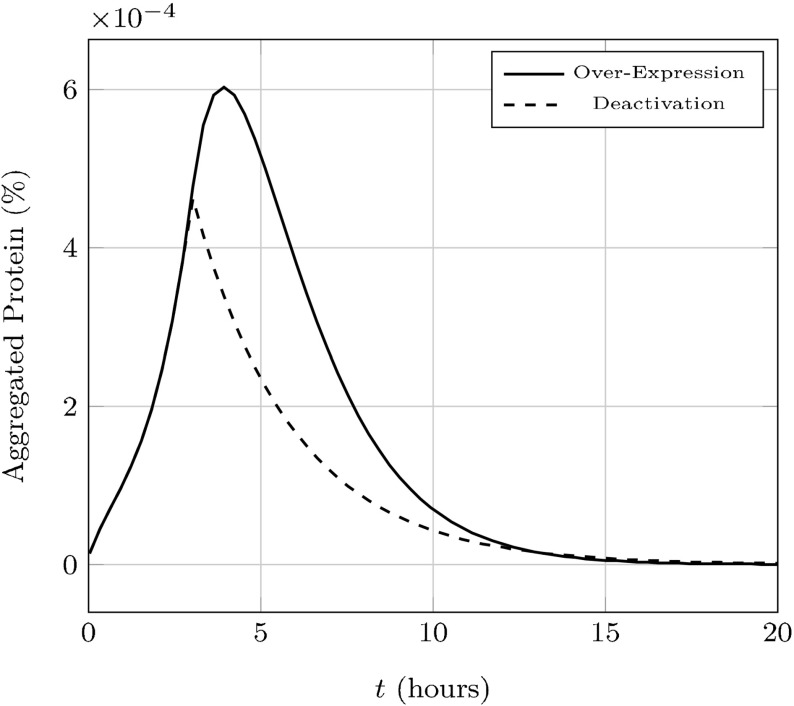
Hsp104 production is up-regulated or Hsp104 is deactivated after 3 h, both by a factor of $10^4$. The new system in either case is unable to stably support the presence of prion aggregates with our engineered parameters

Quantitatively, we treat the inactivation of Hsp104 as letting $k_{\text {on}} \rightarrow 0$. In the limit, we’ll obtain $p'=-k_1p + p^2 - \frac{Bsp}{\omega +n_0-1} < -\frac{Bsp}{\omega +n_0-1} \le 0 \implies p\rightarrow 0^+$. With $p=0$, we have $\eta '=-A_0\eta \le 0 \implies \eta \rightarrow 0^+$, which corresponds to the elimination of prion aggregates. We demonstrate this in Fig. 7.

While earlier studies seemed to indicate over-expression of Hsp104 could cause loss of the prion state (Chernoff et al. 1995), more recent experimental evidence indicates that Hsp104 over-expression is not sufficient to drive prions to extinction (Klaips et al. 2014). In our formulation over-expressing Hsp104 drives $p \rightarrow 1$. This is readily demonstrated by assuming $k_1h = 1/\epsilon \gg 1$ and rescaling time by this quantity; then $p' = 1-p + O(\epsilon )$. However, as is experimentally, this alone is not mathematically sufficient to cure the prion state.

**Fig. 8:**
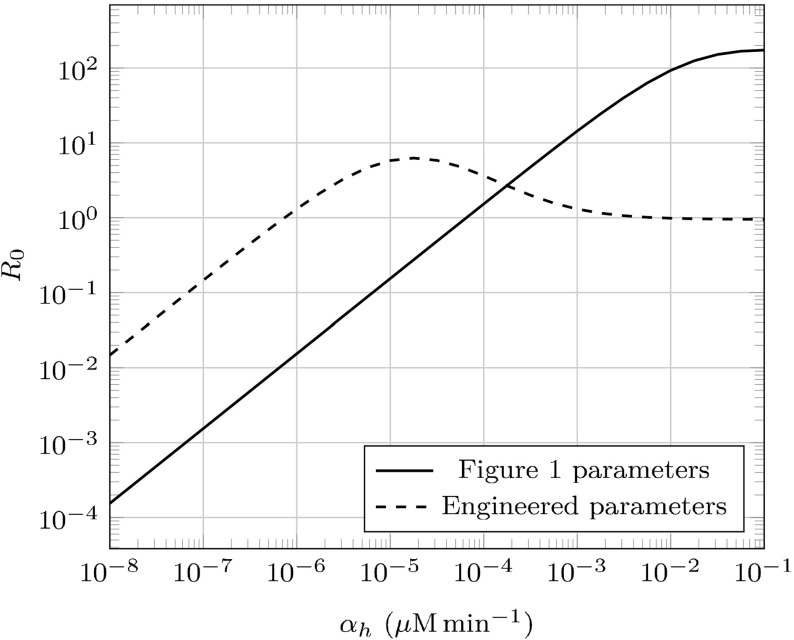
The reproductive number $R_0$ as a function of $\alpha _h$. Prion strains will only be driven to extinction by Hsp104 over-expression if $\lim _{\alpha _h \rightarrow \infty } R_0(\alpha _h) < 1$

Consider an endemic state with $R_0(\tilde{p})=\frac{B/\tilde{p}}{(A_0/\tilde{p}+n_0) (A_0/\tilde{p}+n_0-1)}>1$. Depending on the other kinetic parameters, $R_0$ may be either increasing or decreasing with respect to $\tilde{p}$—prion extinction would only occur if $R_0(1)<1$. Specifically, ${\mathrm {sgn}}(R_0'(p)) = {\mathrm {sgn}}(A_0^2-(n_0-1)n_0p^2)$. If $A_0>n_0-1$, $R_0$ is always increasing and extinction is impossible under our model. This is consistent with the belief that there are other (unmodeled) factors more likely to contribute to prion phenotype loss (Palmer et al. 2011; Klaips et al. 2014); nonetheless, we do provide an engineered parameter set (described in Table 1) that demonstrates prion extinction by maximizing fragmentation efficiency in Fig. 7. We additionally plot the dependence of $R_0$ on $\alpha _h$ in this parameter set as well as the original set we’ve used in Fig. 8.

### Stability of the coendemic prion strains

Up until this point we have considered the aggregates of a single prion species; however, a prion protein is capable of adopting a host of misfolded confirmations each of which is capable of the biochemical processes of conversion of normal protein and fragmentation (Sindi and Serio 2009; Tuite and Cox 2003; Tuite and Serio 2010). That is, $[$*PSI*${}^{+}]$ or PrP$^\text {Sc}$ does not refer to a single prion phenotype, but many related ones, each characterized by different pathology (implying different kinetic parameter values). These distinct prion states are referred to as prion “strains.”

Biologists have observed multiple coexisting strains (Strbuncelj 2009; Polymenidou et al. 2005), but there has been limited mathematical modeling of multiple prion strains. Previously, Tanaka et al. (2006) considered the NPM, under the continuous relaxation of aggregate sizes, with $n_0 = 1$ and demonstrated that if two strains were present then, asymptotically, one strain would dominate and drive the other to extinction. The outcome was determined by the strain which had maximized $\beta \gamma$ (which is proportional to the reproductive number in the case of continuous-size, $n_0=1$ NPM). Since level curves of $\beta \gamma$ represent a set of measure 0 in parameter space, realistically this prevents asymptotic prion strain coexistence. By coexistence, we mean that there exists $i \ne j$ such that $\lim _{t\rightarrow \infty } \eta _i(t), \eta _j(t) > 0$ when $\eta _i(0), \eta _j(0) > 0$ (where $\eta _i$ is the concentration of aggregates of strain *i*).

We now generalize the ELNPM to include multiple prion strains, each capable of converting the same normal protein. Because aggregation is based on conversion to a particular prion strain conformation, we consider an aggregate as consisting of misfolded protein of a single strain. We write the equations for *k* strains with similar constants as before, but scale time by $\varGamma = \sum _{i=1}^k \gamma _i$ and write $\varGamma _i=\gamma _i/\varGamma$:

31a $$\begin{aligned} s'&= A_s(1-s) - s \sum _{i=1}^k B_i \eta _i + \sum _{i=1}^k \varGamma _i(n_i-1)n_i p_i\eta _i \end{aligned}$$

31b $$\begin{aligned} h'&= A_h(1-h) + r \sum _{i=1}^k [(\omega _i+n_i-1)\left( k_{i,-1}p_i- k_{i,1} h[1-p_i]\right) \nonumber \\&\quad + (n_i-1)(n_i-2) \varGamma _i p_i^2]\eta _i \end{aligned}$$

31c $$\begin{aligned} \eta _i'&= \left[ \varGamma _i p_i \omega _i - A_i - (n_i-1)\varGamma _i p_i\right] \eta _i \end{aligned}$$

31d $$\begin{aligned} \omega _i'&= B_i s - \varGamma _ip_i(\omega _i+1)\omega _i \end{aligned}$$

31e $$\begin{aligned} p_i'&= k_{i,1}h[1-p_i] - k_{i,-1}p_i - p_i\left( \frac{B_i s}{\omega _i+n_i-1}-\varGamma _i p_i\right) . \end{aligned}$$

Because our mathematical formulation also requires the molecular chaperone Hsp104, this opens up the possibility for an alternative pathway to prion strain coexistence—rather than out-compete solely on conversion $\beta$ and fragmentation $\gamma$, a second strain may be more efficient at sequestering Hsp104 ($k_{\text {on}}/k_{\text {off}}$). Increasing this ratio improves the strain’s own fragmentation efficiency, as well as decreases the other strain’s efficiency by decreasing the amount of available Hsp104.

It is helpful to think of prion strain competition and coexistence in terms of the reproductive numbers described in Sect. 3.3. Intuitively, the strain with the highest reproductive number will dominate and drive others to extinction. As already stated, this is exactly what Tanaka et al. (2006) observed—however, recall that with the NPM, the reproductive number is fixed, so there will not be any dependency on the kind of or number of strains present. Our model, however, has an effective reproductive number dependent on the current fragmentation efficiency. In terms of strain-specific constants, this number is given by

32 $$\begin{aligned} R_i(p_i) = \frac{B_i/p_i}{(A_i/p_i+n_i-1)(A_i/p_i+n_i)}. \end{aligned}$$

**Fig. 9:**
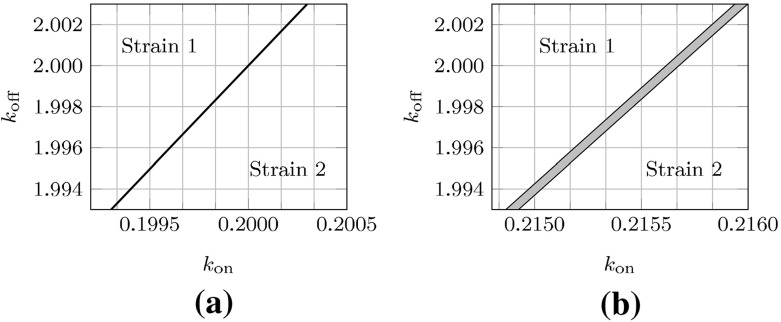
Different parameter regimes exhibit fundamentally different behavior with respect to coendemic stability. The *labeled regions* denote the surviving strain, and *gray regions* denote areas of mutual coexistence. Refer to Table 1 for the parameter values. **a** No stable coexistence, **b** region of stable coexistence

The fragmentation efficiency of strain *i*, $p_i$, is dependent on the current concentration of soluble Sup35 and free Hsp104 (Eq. (31e)), which in turn depend on all of the strains’ concentrations. As such, the reproductive numbers of the strains are coupled to one another. These nonlinear, secondary interactions make analytic determinations of coexistence difficult. However, we are able to numerically demonstrate coexistence of prion strains (Fig. 9).

Remarkably, Fig. 9b demonstrates that strain coexistence is possible for parameters lying in a non-zero area of parameter space. We note that, in contrast to prior models, this type of coexistence is biologically feasible because is it robust to small perturbations in parameter space. Thus, our numerical studies demonstrate that strains with different reproductive numbers (in isolation) can coexist. Further, at the coendemic state each strain’s “cooperative” reproductive number is different from their isolated value, but equal to each of the other strains’ cooperative numbers.

**Fig. 10:**
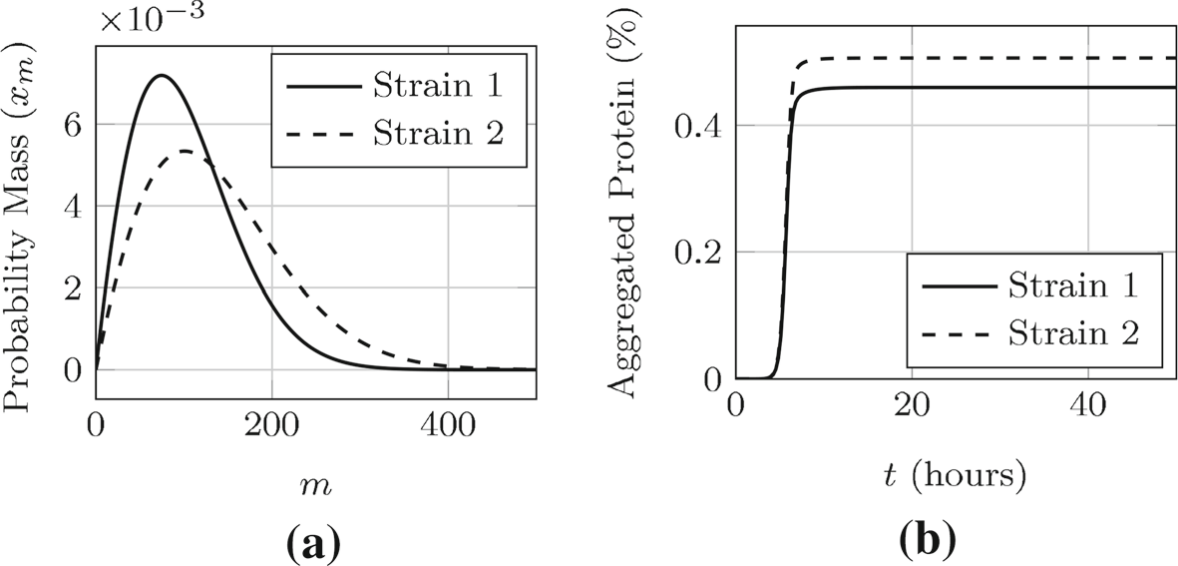
Plots of a specific parameter set admitting asymptotically stable, coendemic behavior. We note that the size densities and aggregated protein take on distinct values, despite very similar reproductive numbers. **a** Steady-state aggregate densities, **b** concentration of Sup35 in aggregates

We choose two specific parameter sets from Fig. 9b and plot of their steady-state, cooperative size densities in Fig. 10a and concentration of aggregated Sup35 in each strain over time in Fig. 10b.

## Conclusion

In this work we successfully developed a mathematical formulation of aggregate dynamics where fragmentation occurs through the molecular chaperone Hsp104. We demonstrate that, under certain restrictions, our model reduces to a numerically tractable form which we call the ELNPM. By including chaperone-mediated fragmentation, this work represents an important step towards a more complete understanding of prion and protein misfolding in vivo.

We derived a unique disease-free steady-state of the ELNPM and analyzed its stability. We demonstrated that the ELNPM supports experimentally observed results such as shifts in the aggregate-size distribution with increasing Sup35 synthesis and response to over- and under- expression of Hsp104. Additionally, it represents a first step towards quantifying prion strain coexistence.

While the ELNPM successfully describes the effects of varying amounts of Sup35 and Hsp104 in the system, we note that there are factors common to enzyme-substrate kinetics that were not included in our model. First, in many biochemical systems there is evidence of cooperation between binding sites (Nelson et al. 2008). Since there is no evidence of interaction between binding sites for Hsp104, we have modeled binding events as purely a function of the free enzyme and available binding sites.

Second, by assuming that $k_{\text {off}}$ was large we were able to assume that for an aggregate consisting of *i* Sup35 monomers with *j* sites bound by Hsp104, all possible configurations of bound Hsp104 are equally likely (see the Supplemental Material). Since under normal expression Hsp104 is observed to be only minimally bound to $[$*PSI*${}^{+}]$ aggregates (Klaips et al. 2014), we interpret this to indicate that $k_{\text {off}}$ must indeed be large relative to $k_{\text {on}}$. Third, we considered a generalization of the uniform fragmentation kernel which corresponds to equality in fragmentation at all binding sites. Together, these three assumptions allowed the use of analytical approaches previously employed in the analysis of the NPM to demonstrate existence, uniqueness and asymptotic stability of the disease-free steady-state.

Beyond Hsp104, other enzymes have been identified as important players in the dynamics of prion aggregate fragmentation (Inoue et al. 2004; Shorter and Lindquist 2008). As such, our mathematical formulation may be taken as the representing collective impact of enzymes on fragmentation. However, compared to the other enzymes, Hsp104 occurs in the lowest molecular number (Ghaemmaghami et al. 2003) and is thus likely to represent a rate limiting step. In addition, we have evaluated our model by comparison to experimental results on the $[$*PSI*${}^{+}]$ prion which has shown to have greatest sensitivity to Hsp104 (Higurashi et al. 2008). Lastly, again note that the form of Hsp104 is a hexamer (Doyle and Wickner 2009)—we have assumed the kinetics of hexamer formation are not relevant to the aggregate dynamics.

In addition to including additional biological complexities, in future studies we plan to investigate global asymptotic stability and explore the conditions underlying prion strain coexistence.

## Electronic supplementary material

Below is the link to the electronic supplementary material.

Supplementary material 1 (pdf 191 KB)
