# Supplementary material for: A mathematical model of the dynamics of prion aggregates with chaperone-mediated fragmentation
Source: J Math Biol. 2015 Aug 22;72:1555–78. doi: 10.1007/s00285-015-0921-0 (PMC4823377; doi:10.1007/s00285-015-0921-0)
Supplement: Supplementary file 1 — Supplementary material 1 (pdf 191 KB) [file 285_2015_921_MOESM1_ESM.pdf]

Supplemental Materials

Jason K. Davis   Suzanne S. Sindi

1 Derivation of ELNPM

1.1 Full Model

We examine the full kinetics implied by the rate equations in Section 2.2, where we consider each possible configuration of bound Hsp104 separately. We define $u_{i(b)}$ to be the density of aggregates of size i with Hsp104 configuration b, where $0 \leq b < 2^{i-1}$ and b's binary expansion is taken to reflect the states of possible fragmentation sites: a 0 appearing in the location of an unbound site, and a 1 in the location of a bound site. Let c_b denote the Hamming weight of b, or the number of 1's appearing in the binary expansion of b. Finally, we assume that fragmentation occurs at a rate proportional to the amount of bound Hsp104 and that the resulting daughters are uniformly distributed amongst the possible configurations. Thus, for $u_{i(b)}$, fragmentation occurs with rate γc_b into daughter aggregates with probability $1/c_b$. Finally, let $b_1 \otimes b_2$ denote the bit-wise and of integers b_1 and b_2 and $\delta(x) = 1$ if $x = 0$ and 1 otherwise. Then,

$$
\begin{aligned}
\frac{du_{m(b)}}{dt} &= -2\beta s(t) u_{m(b)} + \beta s(t) \left[u_{m-1,(b)}\delta(b \otimes 2^{m-2}) + u_{m-1,(b/2)}\delta(b \otimes 1)\right] - (\gamma c_b + \mu_0) u_{m(b)} \\
&\quad + \gamma \sum_{i=m+1}^{\infty} \sum_{k=0}^{2^{i-m-1}-1} \left[u_{i(b+2^{m-1}+2^m k)} + u_{i(k+2^{i-m-1}+2^{i-m}b)}\right] \\
&\quad - k_{\text{on}} h(t) \left[(m-1-c_b) u_{m(b)} - \sum_{\substack{b' \text{ s.t.} \\ c_{b'}=c_b-1 \\ b' \otimes b = b'}} u_{m(b')}\right] + k_{\text{off}} \left[\sum_{\substack{b' \text{ s.t.} \\ c_{b'}=c_b+1 \\ b' \otimes b = b}} u_{m(b')} - c_b u_{m(b)}\right].
\end{aligned} \tag{1}
$$

We now sum over every $b < 2^{i-1}$ such that $c_b = j$, only assuming symmetry in the aggregate configuration densities ($u_{i(b)} = u_{i(b')}$ where b' is the reversed bitstring of b). We write $u_{mn} = \sum_{b \text{ s.t. } c_b = n} u_{m(b)}$ and carefully count bitstrings

and simplify to obtain

$$\frac{du_{mn}}{dt} = -2\beta s(t)\left[u_{mn} - u_{m-1,n}\right] - (\gamma n + \mu_0)u_{mn} + 2\gamma \sum_{i=m+1}^{\infty} \sum_{j=n+1}^{i-(m-n)} \sum_{\substack{b \text{ s.t.} \\ b<2^{m-1} \\ c_b=n}} \sum_{\substack{b' \text{ s.t.} \\ b'<2^{i-m-1} \\ c_{b'}=j-n-1}} u_{i(b+2^{m-1}+2^m b')} - k_{\text{on}}h(t)\left[(m-n-1)u_{mn} - (m-n)u_{m,n-1}\right] + k_{\text{off}}\left[(n+1)u_{m,n+1} - nu_{mn}\right]. \tag{2}$$

We simplify the remaining recovery term with a claim: since conversion effectively biases Hsp104 configurations towards the center of the aggregate, a relatively fast enzyme off-binding will restore the configuration distribution to approximate uniformity. Let us proceed by formally assuming $u_{i(j)} = u_{i(j')}$ if $c_j = c_{j'}$. Then (2) reduces to

$$\frac{du_{mn}}{dt} = -2\beta s(t)\left[u_{mn} - u_{m-1,n}\right] - (\gamma n + \mu_0)u_{mn} + 2\gamma \sum_{i=m+1}^{\infty} \sum_{j=n+1}^{i-(m-n)} \frac{\binom{m-1}{n}\binom{i-m-1}{j-n-1}}{\binom{i-1}{j}} u_{ij} - k_{\text{on}}h(t)\left[(m-n-1)u_{mn} - (m-n)u_{m,n-1}\right] + k_{\text{off}}\left[(n+1)u_{m,n+1} - nu_{mn}\right]. \tag{3}$$

1.2 Reduced Equations

Equipped with these assumptions, let us define the moments of our aggregate density:

$$\eta = \sum_{i=n_0}^{\infty} \sum_{j=0}^{i-1} u_{ij}, \quad z = \sum_{i=n_0}^{\infty} \sum_{j=0}^{i-1} i u_{ij}, \quad z_b = \sum_{i=n_0}^{\infty} \sum_{j=0}^{i-1} j u_{ij}. \tag{4}$$

Then,

$$\frac{ds}{dt} = \alpha_s - \mu_s s(t) - 2\beta s(t)\eta(t) + \gamma(n_0-1)n_0 \sum_{i=n_0}^{\infty}\sum_{j=1}^{i-1} \frac{j}{i-1} u_{ij}(t) \tag{5}$$

$$\begin{aligned}\frac{dh}{dt} &= \alpha_h - \mu_h h(t) - k_{\text{on}} h(t)[z(t)-\eta(t)-z_b(t)] + (k_{\text{off}}+\gamma) z_b(t) \\ &\quad + \gamma(n_0-1)(n_0-2)\sum_{i=n_0}^{\infty}\sum_{j=1}^{i-1}\frac{j(j-1)}{(i-1)(i-2)} u_{ij}(t)\end{aligned} \tag{6}$$

$$\frac{d\eta}{dt} = -\mu_0 \eta(t) + \gamma z_b(t) - 2\gamma(n_0-1)\sum_{i=n_0}^{\infty}\sum_{j=0}^{i-1}\frac{j}{i-1}u_{ij}(t) \tag{7}$$

$$\frac{dz}{dt} = 2\beta s(t)\eta(t) - \mu_0 z(t) - \gamma(n_0-1)n_0\sum_{i=n_0}^{\infty}\sum_{j=0}^{i-1}\frac{j}{i-1}u_{ij}(t) \tag{8}$$

$$\begin{aligned}\frac{dz_b}{dt} &= -(\mu_0+\gamma+k_{\text{off}})z_b(t) + k_{\text{on}}h(t)[z(t)-\eta(t)-z_b(t)] \\ &\quad - \gamma(n_0-1)(n_0-2)\sum_{i=n_0}^{\infty}\sum_{j=1}^{i-1}\frac{j(j-1)}{(i-1)(i-2)}u_{ij}(t)\end{aligned} \tag{9}$$

While simplified, we still lack moment-closure. However, we note that the unclosed terms are of a very particular form, motivating a discrete transformation of u_{ij}. Define

$$v_{mn} = \sum_{j=n}^{m-1} \frac{\binom{j}{n}}{\binom{m-1}{n}} u_{mj}. \tag{10}$$

Then,

$$v_{i,0} = \sum_{j=0}^{i-1} u_{ij}, \qquad v_{i,1} = \sum_{j=1}^{i-1}\frac{j}{i-1}u_{ij}, \qquad v_{i,2} = \sum_{j=2}^{i-1}\frac{j(j-1)}{(i-1)(i-2)}u_{ij}, \tag{11}$$

and more generally,

$$\begin{aligned}\frac{dv_{mn}}{dt} &= -2\beta s(t)\left[v_{mn}(t) - \left(1-\frac{n}{m-1}\right)v_{m-1,n}\right] - \mu_0 v_{mn}(t) \\ &\quad - \gamma(m-n-1)v_{m,n+1}(t) + 2\gamma\sum_{i=m+1}^{\infty} v_{i,n+1}(t) \\ &\quad - n\left[(k_{\text{on}}h(t)+k_{\text{off}}+\gamma)v_{mn}(t) - k_{\text{on}}h(t)v_{m,n-1}(t)\right].\end{aligned} \tag{12}$$

This recurrence is 2nd order in n, so we see that it is ill-posed. We can argue 1 boundary condition without additional assumptions: since $v_{m,n+1} \leq v_{mn}$ by construction, and prion aggregates biologically must have an upper size limit, we have $\lim_{n\to\infty} v_{mn} = 0$. We are now left to find 1 more condition to make the problem well-posed.

1.3 Approximation

We perform the same non-dimensionalization as in Equations (16a)-(16e), writing again $\omega(t) = z(t)/\eta(t) - n_0$ and $p(t) = z_b(t)/[z(t) - \eta(t)]$:

$$s' = A_s(1-s) - Bs\eta + (n_0-1)n_0 \sum_{i=n_0}^{\infty} v_{i,1} \tag{13}$$

$$h' = A_h(1-h) + r\left[(\omega + n_0 - 1)(k_{-1}p - k_1 h[1-p])\eta + (n_0-2)(n_0-1)\sum_{i=n_0}^{\infty} v_{i,2}\right] \tag{14}$$

$$\eta' = (-A_0 + p(\omega + n_0 - 1))\eta - 2(n_0-1)\sum_{i=n_0}^{\infty} v_{i,1} \tag{15}$$

$$\omega' = Bs - p(\omega+1)\omega + (n_0-1)(2\omega + n_0)\left(\frac{1}{\eta}\sum_{i=n_0}^{\infty} v_{i,1} - p\right) \tag{16}$$

$$p' = k_1 h(1-p) - k_{-1}p + p^2 - \frac{Bsp}{\omega + n_0 - 1} + \frac{(n_0-1)(n_0-2)}{p\eta(\omega + n_0 - 1)}\left(p\sum_{i=n_0}^{\infty} v_{i,1} - \sum_{i=n_0}^{\infty} v_{i,2}\right). \tag{17}$$

Written in this way, it becomes clear that enforcing $\sum_{i=n_0}^{\infty} v_{i,n+1} = p\sum_{i=n_0}^{\infty} v_{i,n}$ will yield moment closure. Interpreting u_{ij}/η as a probability mass and (I, J) as a joint random variable modeling aggregate size and bound Hsp104, we write $p = \mathbb{E}[J]/\mathbb{E}[I-1]$ and ultimately understand the nature of our approximation to be the validity of the approximations

$$\mathbb{E}\left[\frac{J}{I-1}\right] \approx \frac{\mathbb{E}[J]}{\mathbb{E}[I-1]}, \tag{18}$$

and

$$\mathbb{E}\left[\frac{J(J-1)}{(I-1)(I-2)}\right] \approx \left(\frac{\mathbb{E}[J]}{\mathbb{E}[I-1]}\right)^2. \tag{19}$$

Consider the 2nd order Taylor expansion of an arbitrary function $f(x, y)$ about $(\bar{x}, \bar{y})$:

$$\begin{aligned} f(x,y) \approx{} & f(\bar{x},\bar{y}) + (x-\bar{x})f_x(\bar{x},\bar{y}) + (y-\bar{y})f_y(\bar{x},\bar{y}) \\ & + \frac{1}{2}\left[(x-\bar{x})^2 f_{xx}(\bar{x},\bar{y}) + 2(x-\bar{x})(y-\bar{y})f_{xy}(\bar{x},\bar{y}) + (y-\bar{y})^2 f_{yy}(\bar{x},\bar{y})\right] \end{aligned} \tag{20}$$

Treating X and Y as random variables and $\bar{x}$ and $\bar{y}$ as their means, then

$$\mathbb{E}[f(X,Y)] \approx f(\bar{x},\bar{y}) + \frac{1}{2}\left(f_{xx}(\bar{x},\bar{y})\mathrm{Var}[X] + 2f_{xy}(\bar{x},\bar{y})\mathrm{Cov}[X,Y] + f_{yy}(\bar{x},\bar{y})\mathrm{Var}[Y]\right). \tag{21}$$

In our case $X = J$, $\bar{x} = z_b/\eta$, and $Y = I - 1$, $\bar{y} = z/\eta - 1$. Let $\bar{u} = z/\eta$; for Approximation (18), we have $f(x, y) = x/y$ and

$$\mathbb{E}\left[\frac{J}{I-1}\right] = p + \frac{1}{\bar{u}^2}\left(p\mathrm{Var}[I-1] - \mathrm{Cov}[J, I-1]\right) + O(1/\bar{u}^3). \tag{22}$$

For Approximation (19), we have $f(x, y) = \frac{x(x-1)}{y(y-1)}$ and

$$\mathbb{E}\left[\frac{J(J-1)}{(I-1)(I-2)}\right] = p^2 - \frac{p(1-p)}{\bar{u}} + \frac{\mathrm{Var}[J] - 4p\mathrm{Cov}[J, I-1] + 3p^2\mathrm{Var}[I-1] - 2p(1-p)}{\bar{u}^2} + O(1/\bar{u}^3). \tag{23}$$

Assuming the variances are dominated by the average aggregate size, we have error terms in the first approximation of $O(1/\bar{u}^2)$ and $O(1/\bar{u})$ in the second; when multiplied against the terms' coefficients, we obtain $O(1/\bar{u}^2)$ in either case.
